# Supplementary material for: Strategic Approach to Massive Chylous Leakage after Neck Dissection
Source: Healthcare (Basel). 2021 Mar 31;9(4):379. doi: 10.3390/healthcare9040379 (PMC8067092; doi:10.3390/healthcare9040379)
Supplement: Supplementary file 1 [file healthcare-09-00379-s001.pdf]

Table S1. Newcastle–Ottawa scale (NOS)

Cases	Selection				Comparability		Outcome			Score
	Is the case definition adequate	Represen tativeness of the cases	Selection of Controls	Definition of Controls	Main factor	Additional factor	Ascertain ment of exposure	Same method for cases and controls	Non response rate	
Eufinger	O	O	-	-	O	O	O	-	O	6
Su	O	O	-	-	O	O	O	-	O	6
de Gier	O	O	-	-	O	O	O	-	O	6
de Gier	O	O	-	-	O	O	O	-	O	6
Wilkerson	O	O	-	-	O	O	O	-	O	6
Gunnlaugsson	O	O	-	-	O	O	O	-	O	6
Ilczyszyn	O	O	-	-	O	O	O	-	O	6
de Gier	O	O	-	-	O	O	O	-	O	6
de Gier	O	O	-	-	O	O	O	-	O	6
de Gier	O	O	-	-	O	O	O	-	O	6
Van Goor	O	O	-	-	O	O	O	-	O	6
Van Goor	O	O	-	-	O	O	O	-	O	6
Chen	O	O	-	-	O	O	O	-	O	6
Casler	O	O	-	-	O	O	O	-	O	6

Is the case definition adequate? We assess whether the definition of case is clear due to the complications of CL caused by cervical lymphatic dissection.

Representativeness of the cases? We evaluate whether this case can represent a case with CL>1 L/day.

Selection of Controls? Not applicable.

Definition of Controls? Not applicable.

Main factor? We evaluate whether the maximum daily CL amount in this case is clearly described.

Additional factor? We evaluate whether there is a clear description of the method and process of handling CL in this case.

Ascertainment of exposure? We assess whether the treatment results of this case are clearly stated, such as survival or death or complications.

Same method for cases and controls? Not applicable

Non response rate? We evaluated whether the time required for complete remission of CL after receiving treatment in this case is clearly stated.

Score: Studies with NOS values > 6 (maximum: 9 points) are considered to be of high quality.
